# Chlamydial conjunctivitis presenting as pre septal cellulitis

**DOI:** 10.1186/1746-160X-3-16

**Published:** 2007-03-14

**Authors:** Suzannah R Drummond, Charles JM Diaper

**Affiliations:** 1 Department of Ophthalmology, Southern General Hospital, Glasgow, Scotland. UK

## Abstract

Chlamydia conjuctivitis results from infection by chlamydia trachomatis, the commonest treatable sexually transmitted infection in Europe. Its clinical manifestations involve the conjunctiva and the cornea. The inflammation under the upper eyelid may be sufficient to present as ptosis, however previously it has not been documented to cause a preseptal cellulitis. We present such a case.

A 15-year-old girl was diagnosed with a left viral conjunctivitis. Five days later, she returned with marked oedema of the left upper and lower lids accompanied by erythema. The tarsal conjunctiva revealed follicles and large papillae and extra ocular movements revealed discomfort on elevation. A secondary diagnosis of bacterial pre septal cellulitis was made and the treatment was changed a broad spectrum oral antibiotic. On review at two days, the patient now complained of a large amount of purulent discharge in association with the marked pre septal swelling. As previous bacteriology and virology had been negative, the patient was re swabbed for chlamydia. This proved positive and her symptoms completely resolved following administration of Azithromycin.

In this particular case recognition of the pathogen is important to alert the patient to the likelihood of unknown genital infestation. In all cases of positive culture, the patient should be counselled to attend a genitourinary clinic and to alert any sexual partners to the need to do likewise.

## Background

Chylamydia trachomatis is the commonest treatable sexually transmitted infection in Europe. There is a10 % prevalence in women aged 16–24 years attending UK pregnancy or genitourinary services [1].

Chlamydia (or adult inclusion) conjunctivitis is the most common cause of chronic follicular conjunctivitis resulting from infection by Chlamydia trachomatis. It commonly manifests as a unilateral or bilateral asymmetric conjunctivitis associated with moderate hyperemia and mucopurulent discharge. It predominates in young, sexually active adults.

Clinical manifestations of the conjunctivitis involve the conjunctiva and the cornea. The inflammation under the upper eyelid may be sufficient to present as ptosis. However, previously it has not been documented to cause such a degree of swelling and inflammation of both the lids to warrant a diagnosis of preseptal cellulitis. We present such a case.

## Case Report

A healthy 15-year-old girl was referred following a five day history of a unilateral red left eye. The eye was becoming progressively more inflammed, with epiphora, photophobia and blurred visual acuity.

On examination, the visual acuity in the affected eye was 6/6 compared with 6/5 in the other eye. The conjunctiva was inflamed with a follicular reaction including the corneal margins superiorly. There were enlarged pre auricular nodes. A diagnosis of viral conjunctivitis was made and viral plus bacterial swabs were taken.

The patient was commenced on fucithalmic to prevent secondary infection and told to re attend if she deteriorated. Five days later, she returned feeling that the eye had become more tender with increased swelling of the lids plus tenderness over the maxillary sinus.

On examination, there was marked oedema of the upper and lower lids accompanied by erythema. The tarsal conjunctiva revealed follicles. Extra ocular movements were full but uncomfortable on elevation. Pupil, colour vision examination and direct visualisation revealed a healthy disc. She was apyrexial and systemically otherwise well. A secondary diagnosis of bacterial pre septal cellulitis was made and the treatment was changed to oral ciprofloxacin 750 mg twice daily for one week, plus two hourly topical exocin drops.

Two days later the patient was reviewed. She now complained of a large amount of purulent discharge. The pre septal swelling was still marked and examination of the conjunctiva again revealed large numbers of follicles and large papillae.

All bacteriology and viral swabs had been negative and the patient was re swabbed for chlamydia despite denying any genitourinary symptoms. Giemsa staining of conjunctival scrapings revealed cytoplasmic inclusion bodies and the patient received a one gram single dose of Azithromycin. She was asked to attend the local genitourinary clinic and to alert any current and previous sexual partners to their need to do likewise. Her symptoms completely resolved following administration of the Azithromycin and there were no further complications. Unfortunately, the patient declined to have photographs taken.

## Comment

Preseptal Cellulitis may occur in three clinical scenarios [2]; as a direct result of localised trauma, as an infection or inflammation of adjacent structures or in patients with coexisting sinusitis. H. Influenza, Pneumococcus and Staphylococcus species are all commonly implicated in the disease [3] but other pathogens including atypical bacteria [4] and fungi [2] are responsible for a minority of infections. It is difficult to determine the pathogen responsible for any cellulitis without aspirating a culture sample and so treatment is usually instituted by an assumption of the most common causative organisms. [5]

There are no specific features which point to the infection being from an atypical organism rather than a more common pathogen. However, it is important that the physician pays particular attention to the combination of history and signs during the consultation. In this case, the symptoms were present in a sexually active young female with concurrent evidence of a papillary conjunctivitis productive of purulent discharge.

Chlamydial conjunctivitis results from accidental transfer of genital discharge infected with *Chlamydia trachomatis*, an obligate intracellular parasite, into the eye. C Trachomatis infects moist mucosal surfaces producing covert damage principally by triggering a localised cell-mediated immune response which is magnified by repeated exposure to infection. [1]

The hallmark signs include conjunctival injection with large inferior follicles and a superior papillary reaction. Commonly, the condition will have persisted for over three weeks despite treatment with topical antibiotics. Unlike common viral conjunctivitis, chlamydia infection tends to affect the cornea in terms of peripheral subepithelial infiltrates and diffuse superficial punctuate keratitis. It can also cause superior corneal pannus, corneal ulceration and iritis. A palpable pre auricular node is almost always present.

The inflammation under the upper eyelid may present as ptosis due to the increased weight of the inflamed tissues. However, previously it has not been documented to cause such a degree of swelling and inflammation of both the lids as to lead to preseptal cellutitis.

Preseptal cellulitis – if left untreated has the potential to cross the septal barrier and spread to the posterior orbit resulting in fatal complications. In addition however, in this particular case the importance of recognition of the pathogen is to alert the patient to the likelihood of unknown genital infestation. Most genital infections are asymptomatic and thus the disease is endemic. As well as the potential urogenital complications including ectopic pregnancy and salpingitis which may lead to infertility, it is also associated with other serious non genital manifestations including perihepatitis.

In all cases of positive culture, the patient should be counselled to attend a genitourinary clinic and to alert any previous sexual partners to the need for testing and treatment.
